# Dr. Edward Brown-Sequard’s Experimental and Clinical Researches Applied to Physiology and Pathology

**Published:** 1857-03

**Authors:** 


*Dr. Edward Brown-Sequard's Experimental and Clinical Researches applied to Physiology and Pathology.*

*Fourth*—Cases of Cure of Epilepsy by Cauterization and other Local Means of Modification of the Parts from which originates the Aura Epileptica.

There are a great many cases of this kind. They bear out the same conclusion as the cases of section of a nerve, in showing that the fits were caused by a peculiar influence originating from some part of the skin. Cauterization of the skin of the face and neck by the red-hot iron, in my animals, seems to cure them, as I will show hereafter. It appears, therefore, that there is something of the same kind in the condition of the skin of the neck and face in these animals, and in the parts of the skin which are the seat of a true aura epileptica in man.

The most varied modes of cauterization have been employed with success against the aura epileptica. Blisters, moxas, potential cauteries, issues, Dippel's oil, a decoction of ruta graveolus, and various other rubefacients have been successful in cases reported by Locher, Baster, Dovinetus, Brunner, Stuerlin, Henricus ab Heer, Benzi, Portal, Recamier, &c.

It is useless to mention any of these cases particularly, because there are so many on record that every one knows some of them.

The application of a moxa or of the red-hot iron, is, I believe, the best means of cauterization—at least it is so for animals: and the many cases in which epileptics have been cured by a burn (see Portal, *loco cit.* pp. 160 and 172), agree in showing the power that burning of the skin possesses. In a case by Tulpius (see Herpin, *loco cit.* p. 399), the aura came from the big toe, and the patient was cured by deep burnings of this toe with the red-hot iron.

Any kind of change in the skin may be the cause of the appearance of epilepsy or of its disappearance. A man, says Esquirol (*loco cit.* p. 304), had an ulcer on one of his legs; epilepsy came on after the cicatrization of the ulcer, and each fit was preceded by the sensation of a cold wind in the cicatrix; a ligature above the knee-joint stopped the fit. A young man, whose case is recorded by Pouteau (quoted by Portal, *loco cit.* p. 375), had received a blow on the head, and the wound was cicatrized only a year after; he was then attacked with epilepsy, and the fits gradually became more and more frequent. After having been a year in this condition, he consulted Pouteau, who opened the cicatrix by the application of the cautery. From this day the fits disappeared; but the patient allowed the wound to be healed again, and epilepsy returned. It disappeared again, after another application of the caustic.

Perhaps various operations which have been followed by the cure of epilepsy, are to be explained in the same way as the many cases related in this paragraph. This is true, perhaps, for a case mentioned by Delasiauve (*Traite de l' Epilepsie*, p.

430), and in which, after the extirpation of an encephaloid tumor in the angle of the jaw, an epileptic patient was cured. This explanation is probably good, also, for some of the cases in which trepanning of the cranium has been successful in epileptic patients. Among the cases of this kind that I know, I take four, almost at random, to show the fitness of this explanation. In one of them, a circumscribed and permanent pain in the head, led Dr. James Guild to apply the trephine. The patient was cured—(Delasiauve, *loco cit.* p. 422). In another case, Dr. Campbell (*Annales Med-Psychol.* Vol. XIII. p. 613) applied the trephine on the cranium of a man who had received a blow, and who suffered a great deal from the wound it had produced. No more fits took place, and four years after the operation the man was still well. In a third case, recorded by Benjamin Travers (*A further Inquiry concerning Constitutional Irritation and the Pathology of the Nervous System*, p. 285), the trephine was applied in a place where the cranium was depressed and painful to the touch. The patient was cured. The fourth case I will give in full, as it has not yet been published, and also on account of its importance. I owe the history of this case to Professor Van Buren, of New York, and I give it just as it has been furnished to me by this distinguished surgeon:—

*Case IX.*—'A healthy married woman, twenty-six years of age, received a blow upon the side of her head from the clenched fist of her husband, who was intoxicated. The seat of the injury remained permanently tender to the touch, and about five months afterwards she had an epileptic fit for the first time. The fits recurred from this time in gradually diminishing intervals, and when she was admitted into the New York Hospital, in March, 1856, about three years after the injury, they occurred almost every day.

'Over the centre of the parietal bone of the right side, a portion of the scalp, about the size of a half dollar, was very sensitive on pressure, but no appreciable lesion could be discovered, except, perhaps, a slight puffiness of the integuments at this point. She suffered much from headache, the pain always commencing here, and seeming to radiate from this tender surface to the rest of the head. Before a seizure of epilepsy this local pain, which was always present, invariably became more intense.

'After watching the patient for some weeks, during which time the fits were evidently becoming more frequent, it was observed that she was worse at her catamenial period. In fact, upon the 5th and 6th of April she had no less than twenty-seven distinct seizures. Her memory and other intellectual faculties were observed to be decidedly impaired. In other respects her health was good. Valerianate of zinc was tried in doses of two and three grains three times a-day during a fortnight, but without benefit.

'It was then decided, in consultation, to explore the condition of the scalp and cranial bone at the seat of pain, and to remove a portion of the bone, if it showed any evidences of disease. This was done on the 10th of May. The patient was etherized, and a free crucial incision made through the scalp. The periosteum was found more than naturally adherent to the bone, the surface of which was somewhat elevated and roughened over a space an inch and a half in diameter. This altered portion of bone was removed by two applications of the trephine; its inner surface was found to be perfectly normal, but its diploe was obliterated.

'The wound was closed accurately, except at the point where the incisions crossed, and cold water dressings applied. No fit occurred until the 18th of May, when she had three during the day and evening, followed by active febrile symptoms with nausea, and on the following day an erysipelatous blush appeared upon the forehead. On the 19th and 20th she had three fits, but they were not very severe. The attack of erysipelas lasted the usual time, and proved to be rather a severe one. The wound of the scalp healed kindly and uninterruptedly, and at the end of the erysipelas was entirely cicatrized (May 27th). After the seizure, which occurred on the 20th, there was no return of the epilepsy. The patient was retained in the Hospital until after a menstrual period, and as this did not take place at the usual time appropriate remedies were employed, but it was not until the sixth week that the catamenia returned, so that the patient was not discharged from the Hospital finally until July 10th, having had no fit meanwhile.

'The epileptic fits which occurred on the 18th, 19th, and 20th of May, coincidently with the invasion of the erysipelas, seem to have taken the place of the usual chill, as her attack commenced without one; and they were the only fits which occurred after the operation of May 10th.

'I have seen the patient twice since her discharge from the Hospital, once within the past month (November), and she is in perfect health, having had no threatening whatever of an epileptic fit since those which ushered in the attack of erysipelas.'

The extirpation of two pieces of altered bone in this case has certainly not been the cause of the cure of the patient, as there have been fits after their removal. We are led, therefore, to admit that the cure was the consequence either of the influence of the erysipelas or of a change that took place in the skin while the wound was healing. There are cases on record where either erysipelas, or some other febrile disease, seems to have cured epilepsy; but this is so very rare, that it is much more probable that in the patient of Dr. Van Buren the cure has been effected by the change that the operation has produced in the skin, just where the blow which had caused the epilepsy had been received. The frequency of cures of this convulsive disease by anything that may produce a change in a part of the skin, which, being injured or the seat of a pain, has caused epilepsy, renders it very probable that in this case the cure has been obtained by the change produced by the operation.

While I think that Dr. Van Buren deserves great eulogy for this bold and successful operation, I nevertheless ought to say, that with the knowledge I have now that epilepsy originates very frequently in the skin, it would be necessary in the future, in cases like those I have just recorded, to employ various means of cauterization, and particularly the application of a red-hot iron upon the injured skin before making use of the trephine. Very likely cauterization, in a number of cases, will prove sufficient to cure.

Perhaps we are authorized to place the cases we will speak of now, among those in which the skin was a source of an aura epileptica.

J. Carron (*Journal General de Medecine*, Vol. XIII. p. 242) relates the following case:—

*Case X.*—A child, eleven years old, had fits of epilepsy two or three times a week, since he was two years old. A feeling of cold, coming from one of the upper extremities, preceded the fits. A ligature having been applied around the arm and tightened at each threatening, the fits were avoided. A small tumor was then found on the first phalanx of the thumb, and to ascertain if this tumor was the cause of the fits, although it did not produce pain, the ligature was placed successively on the hand and on the thumb, and the fits were prevented. An incision was then made upon the tumor, and four very small bodies of hard sebaceous matter were taken out. The wound was excited to give much pus, and healed after thirty days. The child was completely cured, and has never had a fit since.

Portal (*Anatomie Medicale*, Vol. IV. p. 247,) gives the case of a woman whose fits began by a pain in the thumb. Leduc, a pupil of Portal, extirpated a hard portion of the skin (a bunion, very likely—*un durillon*), and the patient was cured.

A strange body in the ear had caused epilepsy. Fabricius Hildanus extirpated it, and the patient was cured. (Esquirol, *loco cit.* Vol. I. p. 303).

Esquirol says (*loco cit.* Vol. I. p. 303): 'Donat attended a nun who felt, in the beginning of the fits, a pain in the right mamma, from which the aura ascended to the brain: 'If an ulceration took place in the mamma the fit was prevented.'

Although the skin is more apt to produce epilepsy than the trunks of nerves, there are many cases where an injury to the trunk of a nerve has caused this disease. Such cases have been recorded by De Haen, Henning, Larrey, Romberg (*Nervenkrankheiten*, 3d ed. Vol. I. part 2, p. 689) and others. I will relate some cases of this kind to show that for them, as for those in which the aura epileptica originates in the skin, the same principle is true, that an interruption between the injured part and the brain is able to cure epilepsy.

Portal (*Observ. sur l' Epilepsie*, p. 210) gives the case of a man who had a nerve injured in the arm. Convulsions, with loss of consciousness, came on many times. A greater incision was made where the wound existed, and the patient was cured.

The same writer (*loco cit.* p. 156) speaks of a man who had received a pistol shot in the neck, and who had become epileptic. After some time an abscess was formed in the neck; one of the shot came out, and the patient was cured.

Dieffenbach (*Die Operative Chirurgie*, Vol. I. p. 852) relates the case of a young girl, whose hand had been wounded by a piece of bottle-glass. Neuralgic pains, epileptic fits, and contraction of the limb, had been the results of the wound. The cicatrix was opened, and a small bit of glass was found near a nerve which had been divided by it, and which was swollen and hardened. After the operation, the neuralgia, the epilepsy, and the contraction vanised, and the girl was completely cured.

Fizes, according to Portal (*loco cit.* p. 157), has seen a man who had become epileptic after having been wounded by a sword near the great angle of the eye, and who was cured after the extirpation of a small part of the point of the sword which had staid in the wound.

Cases, more or less resembling the preceding, have been reported by Lamotte, Van Swieten, Sauvages, De Haen, Burserius, Lamorier, &c.

Darwin reports that he once saw a child who frequently fell down in convulsions. A wart was found on the ankle, which was cut off, and the fits never recurred.

Epilepsy caused by the irritation of the dental nerves, and cured by the extirpation of some teeth, or by the lancing of the gums, is not uncommon. Some interesting cases of this kind have been reported by Portal (*loco cit.* p. 205 and elsewhere).

I shall not speak here of the cases of epilepsy produced by an irritation of a mucous membrane, or of a viscus, and which have been cured by the removal of the irritation. These cases are very numerous, and they also prove that epilepsy may be cured by the suppression of the irritation of nerves, either in their peripheric ramifications or in their trunks.—*Boston Med. and Surg. Journal.*

